# Data set for demography, clinical characteristics, and risk factors of trigeminal neuralgia patients in Addis Ababa, Ethiopia

**DOI:** 10.1186/s13104-020-05425-3

**Published:** 2021-01-06

**Authors:** Biniyam A. Ayele, Yared Zenebe Zewde, Mikyas Tilahun Degefa, Abenet Tafesse Mengesha

**Affiliations:** 1 Department of Neurology, School of Medicine College of Health Sciences, Addis Ababa University, Po Box 6396, Liberia Street, Lideta sub-city, Addis Ababa, Ethiopia; 2 Amanuel Mental Specialized Hospital, Addis Ababa, Ethiopia

**Keywords:** Trigeminal neuralgia, Data, Characteristics, Risk factors, Ethiopia

## Abstract

**Objectives:**

Pain is one of the most ignored clinical symptoms in resource limited country such as Ethiopia. Trigeminal neuralgia (TN) is one of the most painful medical illnesses known to human. Very little was reported about TN from the sub Saharan Africa (SSA), especially from Ethiopia. We aimed to study the demographic, clinical characteristics, and risk factors of TN in sixty-one patients at two public and two private health facilities in Addis Ababa Ethiopia. These data will be vital to researchers and clinicians interested in knowing the pattern of TN in SSA in order to compare and contrast with similar data from the west.

**Data description:**

The data set contains characteristics of TN patients. All the variables in the data set were coded by self-explanatory codes. The data set contains: demographic data, which contains age ranges and duration of illness; clinical characteristics data contains clinical characteristics, and risk factors includes such as structural brain abnormalities, family history, and dental extraction. Nearly 90% of the patients had Classical TN. The right side and mandibular branch of trigeminal nerve was commonly involved. Close to 40% reported previous tooth extraction history. Majority of the patients reported satisfactory pain control with carbamazepine.

## Objective

Pain is one of the most ignored clinical symptoms in resource limited country such as Ethiopia. Likewise, Trigeminal neuralgia (TN) is one of the most painful medical illnesses known to human [1]. However, very little was reported about TN from the sub Saharan Africa (SSA), especially from Ethiopia. Therefore, we aimed to study the demographic pattern, clinical characteristics, and risk factors of TN in sixty-one patients at two public and two private health facilities in Addis Ababa Ethiopia. This data set was stored in an online data repository and will be vital to researchers, clinicians, and dental doctors who are interested in knowing the pattern of Trigeminal neuralgia in SSA country in order to compare and contrast with similar data from the western countries [2].

**Table 1:** Overview of data files/data sets

Label	Name of data file/data set	File types (file extension)	Data repository and identifier (DOI or accession number)
Data file 1	TN.data_set.svc.sav	.sav	Mendeley http://dx.doi.org/10.17632/7w4hsm63nk.5
Data file 2	TN questionnaire revised.docx	Word document	Mendeley http://dx.doi.org/10.17632/7w4hsm63nk.5

This data was collected between June 2019 and March 2020 at four health facilities in Addis Ababa, Ethiopia. Initially we planned to enroll patients from two public hospitals. The study received ethical approval from the Institutional Review Board (IRB) at the College of Health Sciences Addis Ababa University (Protocol No. 043/19/Neuro). All subjects provided written consent before conducting the interview. However, because of the COVID 19 pandemic the number of non-COVID neurological patients plummeted in the two public hospitals. Therefore, we decided to include two private health facilities with relatively good neurological case load in order to complete our survey. All the sixty-one patients’ were diagnosis with TN as per the International Classification of Headache Disorders-3rd Edition (ICHD-3) criteria by certified neurologist who are part of the research [3]. Recently we have published part of this data set including demographic data, duration of illness, clinical characteristics including classification of TN, affected branch of trigeminal nerve, side of face affected, quality of pain, exacerbating factors such as seasonal variation, treatment satisfaction, types of medication used to treat TN, history of dental extraction, and family history of TN. Data related to brain imaging findings, neurosurgical intervention for TN management, and comorbid illness will be submitted for publication soon [4].

## Data description

The data set contains demographic and clinical characteristics of patients with TN diagnosed based on International Classification of Headache Disorders-3rd Edition (ICHD-3) criteria [3]. Trigeminal neuralgia patients often visit dental doctors before their neurologist visits; this is because, the TN-related facial pain often involves trigeminal nerve branches around maxillary and mandibular region. Furthermore, the pain may involve the upper or lower gingiva mimicking dental pains [5]. Therefore, such prolonged latency period between dental and neurologist visit will results in delayed and miss diagnosis. Because of such delayed diagnoses and relatively low prevalence of the disease, it’s difficult to enroll adequate number of patients with TN in our study. However, this is an ongoing research project and we are still actively enrolling new patients with TN in all the study settings.

All the variables in the data set were coded by self-explanatory codes. Two kind of data set was uploaded on online data repository. The first data set contains the main rough data in Statistical Package for the Social Sciences (SPSS) version 25 and the second data set contains the main questionnaire used to extract data during patient’s interview.

Out study was a cross sectional observational study and conducted between June 2019 and March 2020. All patients with confirmed diagnosis of TN were included. Therefore, no sample size was calculated in the present study. Thus, consecutive patients with diagnosis of trigeminal neuralgia were interviewed and examined by certified neurologists.

The first data set contains three category of variables this includes demographic, clinical characteristics, and risk factors. Demographic data includes age ranges and duration of illness. The present data set showed the age range of the study participants were between 21 and 78 years. The average duration of illness was 5.7 (3.9) years. The second data category contains the following variables: classification of TN, presenting features, side of face involved, trigeminal nerve branch involved, triggering factors, triggering zones, quality of pain, types of treatment, treatment satisfaction, neurological examination, seasonal variation of pain, associated autonomic, worsening of pain during sleep. Fifty-five (90.2%) of the patients have classical TN. Majority of the patients reported mixed type of pain including, burning, lancinating, and electric shock type of pain. Mandibular branch of trigeminal nerve was commonly affected nerve branch in our study. No patient had involvement of ophthalmic branch involvement. All the patients had normal neurological examination, except one patient who had objective sensory abnormality on the affected side. Majority of our study participants were treated by carbamazepine only. The average dose of carbamazepine among our study participants was 600 mg (range: 0–2000 mg). Forty-two (68.8%) study participants reported prominent satisfaction with their pain treatment. The third data category was data which includes trigeminal neuralgia risk factors such as brain imaging showing structural brain abnormalities and family history of TN. Out of sixty-one participants forty-nine (80.3%) had brain imaging. Out of forty-nine patients with TN who had brain imaging, 36.1% was unremarkable. Symptomatic TN was observed in small proportion of our study participants. The identified secondary causes include cerebro-pontine angle (CPA) meningioma, brainstem cavernoma, and multiple sclerosis. Among total of sixty-one study participants twenty-five (41%) of the patients reported visit to dental office and removed or filled of one or more lower molar/premolar tooth as a treatment of facial pain secondary to TN [2, 4].

## Limitations

The first short coming of this observational study includes low patients enrollment; this is because of the low prevalence of TN. Furthermore, in low income countries such as Ethiopia, where there are few trained neurologist, early and accurate diagnosis of TN is a major challenge. However, we have decided to keep requiting patients with TN to have an adequate cohort of patients with TN. The second limitation was absence of control group. Controlled studies are generally considered to be source of strong scientific evidences. However, due to lack of financial and trained personnel, we couldn’t able to include controlled group in our study. Therefore, we recommend conducting future controlled study to consolidate our study findings. Finally, the universal lack of advanced imaging investigations such as MRI and MR angiography had hindered our capacity to accurately estimate the burden of symptomatic TN in our patients.

## Data Availability

The data described in this Data note can be freely and openly accessed on http://dx.doi.org/10.17632/7w4hsm63nk.5. Please see Table 1 and reference [2] for details and links to the data.

## Abbreviations

ICDH-3: International Classification of Headache Disorders-3rd Edition
TN: Trigeminal neuralgia
MRI: Magnetic resonance imaging
